# *In vitro* Manganese-Dependent Cross-Talk between *Streptococcus mutans* VicK and GcrR: Implications for Overlapping Stress Response Pathways

**DOI:** 10.1371/journal.pone.0115975

**Published:** 2014-12-23

**Authors:** Jennifer S. Downey, Lauren Mashburn-Warren, Eduardo A. Ayala, Dilani B. Senadheera, Whitney K. Hendrickson, Lathan W. McCall, Julie G. Sweet, Dennis G. Cvitkovitch, Grace A. Spatafora, Steven D. Goodman

**Affiliations:** 1 Division of Biomedical Sciences at the Herman Ostrow School of Dentistry of the University of Southern California, Los Angeles, California, United States of America; 2 Center for Microbial Pathogenesis, The Research Institute at Nationwide Children's Hospital, Columbus, Ohio, United States of America; 3 Dental Research Institute, Faculty of Dentistry, University of Toronto, Toronto, Ontario, Canada; 4 Middlebury College, Department of Biology, Middlebury, Vermont, United States of America; East Carolina University School of Medicine, United States of America

## Abstract

*Streptococcus mutans*, a major acidogenic component of the dental plaque biofilm, has a key role in caries etiology. Previously, we demonstrated that the VicRK two-component signal transduction system modulates biofilm formation, oxidative stress and acid tolerance responses in *S. mutans*. Using i*n vitro* phosphorylation assays, here we demonstrate for the first time, that in addition to activating its cognate response regulator protein, the sensor kinase, VicK can transphosphorylate a non-cognate stress regulatory response regulator, GcrR, in the presence of manganese. Manganese is an important micronutrient that has been previously correlated with caries incidence, and which serves as an effector of SloR-mediated metalloregulation in *S. mutans*. Our findings supporting regulatory effects of manganese on the VicRK, GcrR and SloR, and the cross-regulatory networks formed by these components are more complex than previously appreciated. Using DNaseI footprinting we observed overlapping DNA binding specificities for VicR and GcrR in native promoters, consistent with these proteins being part of the same transcriptional regulon. Our results also support a role for SloR as a positive regulator of the *vicRK* two component signaling system, since its transcription was drastically reduced in a SloR-deficient mutant. These findings demonstrate the regulatory complexities observed with the *S. mutans* manganese-dependent response, which involves cross-talk between non-cognate signal transduction systems (VicRK and GcrR) to modulate stress response pathways.

## Introduction

*Streptococcus mutans*, one of the primary etiological agents of dental caries, can metabolize dietary carbohydrates and produce lactic acid as a fermentative end-product [1]. In addition to its acidogenicity, *S. mutans* is also aciduric, owing partly to an acid tolerance response [2] that allows it to adapt to conditions of low pH in the plaque environment. Part of this adaptive response is facilitated by differential regulation of genes under acid stress that include those whose products mediate proton extrusion (e.g. *atpE/A*), alter membrane composition (e.g. *fabM*, *ffh*) and assist with DNA repair (e.g. *uvrA, recA*) [3]–[8].

In previous work, we defined a role for GcrR (also known as CovR), in the *S. mutans* ATR and noted that *gcrR* expression was subject to metalloregulatory control by SloR [4]. The SloR metalloregulator in *S. mutans* is a DtxR homolog that controls the expression of a plethora of genes in response to metal ion availability, particularly manganese [9]. Manganese is an important micronutrient that has been correlated with streptococcal virulence and caries incidence [10]. The SloR regulon controls manganese-responsive genes that encode sucrose-independent and –dependent adherence (*spaP*, *gbpC*, *gtfB* and *gtfC*, genetic competence (*comD/E*), and oxidative stress tolerance (*sod*), all of which were shown to be up-regulated by SloR [4], [9], [11], [12]. More recently, it was demonstrated that manganese limitation increased *gcrR* expression in a SloR-dependent manner [4].

In contrast with what is found in other closely related streptococci, in *S. mutans gcrR* is not part of a two-component system (TCS) but rather is an orphan response regulator (RR), meaning it is not genetically linked to a cognate histidine kinase [13] [14]. A typical TCS is comprised of a membrane-bound HK, which autophosphorylates when activated by an environmental stimulus and subsequently transphosphorylates a cytosolic RR, which is often co-transcribed as an operon with the HK. The phosphorylation of the RR typically results in its activation, thus facilitating the RRs binding to DNA promoter/operator regions to modulate the expression of genes under the control of the TCS [15]. Unlike in *S. mutans*, the GcrR ortholog in *Streptococcus pyogenes* (designated CovR), is genetically linked to its cognate HK, CovS. In fact, CovRS has been extensively studied in group A streptococci (GAS) and more than 15% of the GAS genome, including those genes that mediate growth, virulence, biofilm formation, and stress tolerance are controlled by the CovRS TCS [16]–[22].

In *S. mutans*, GcrR has been implicated in the stress tolerance response; in particular, GcrR was shown to up-regulate expression of *atpEA* and *ffh* in the ATR mechanism [4]. These genes encode a proton extruding ATPase and a signal recognition peptide that facilitates ATPase insertion into the bacterial membrane, respectively [4], [8]. A clear role for GcrR in the regulation of the bacteria's biofilm phenotype was shown as GcrR modulates *S. mutans* sucrose-dependent adherence through direct binding to the glucosyltransferase B and C (*gtfB* and *gtfC*) promoter regions to repress their transcription [11], [12]. Expression of *gtfBC* is critical for biofilm formation and the pathogenicity of *S. mutans* [23], and therefore is tightly regulated by multiple signal transduction systems at the transcriptional and/or translational levels [24]–[28]. One such system is the VicRK TCS, that has a drastic effect on the biofilm phenotype of *S. mutans*, and was shown to have a positive regulatory impact on *gtf* expression [29].

Of 14 TCSs present in *S. mutans*, the VicRK TCS is noteworthy as the only signaling system that is essential for its viability. VicRK has been shown to be essential for survival and virulence in a wide range of bacteria, including the streptococci, bacilli and the staphylococci [29]–[32]. Depending on the bacterial species, either both VicK and VicR or just VicR alone are essential (the latter is true in *S. mutans*). The VicRK homologs (also known as YycGF and WalKR) in *Staphylococcus aureus* and *Bacillus subtilis* have been implicated as the master regulatory system for cell wall metabolism by positively regulating autolysin synthesis and biofilm formation [32], [33].

Previous work has demonstrated that in addition to modulating the *gtfB, gtfC*, and *gtfD* genes for biofilm formation, *S. mutans* VicRK is also involved in genetic competence development, acid production, cell viability and tolerance of oxidative and acidic stressors in this organism [29], [34]–[39].

Despite these findings, the genetic basis for *S. mutans* VicRK-modulated stress tolerance is not well-understood and the signal(s) that stimulate VicK activation remain unknown. Therefore, an improved understanding of how VicRK modulates these various stress responses through gene expression could provide insight into how bacterial TCSs might be manipulated, thereby fostering the development of therapeutics against bacterial infections.

In the present study, we report that *S. mutans* VicK can transphosphorylate not only its cognate RR, VicR, but also the orphan RR, GcrR; the latter is only demonstrable *in vitro* in the presence of manganese. We also demonstrate that while autophosphorylation and transphosphorylation reactions were enhanced by manganese, the specificity for a given RR appears specific for just VicR and GcrR. In addition, we present evidence to support a role for both VicK and SloR in *gcrR* transcriptional control. Finally, we show that the DNA binding sites of VicR and GcrR overlap in common downstream gene promoters further integrating the two RRs. A model is proposed for the biological basis for cross-talk between VicK, VicR and GcrR.

## Methods

### Bacterial strains, plasmids and growth conditions

The bacterial strains and plasmids used in this study are described in Table 1. *Escherichia coli* was grown overnight at 37°C in Luria–Bertani broth (also known as LB broth, Difco) with gentle aeration, or on Luria-Bertani medium containing 1.5% w/v agar (LB agar). Kanamycin sulfate and ampicillin were added to these media when appropriate, each at a final concentration of 100 µg ml^−1^. *S. mutans* was grown as standing overnight cultures at 37°C and 5% CO_2_ in Todd-Hewitt broth (Becton Dickinson) supplemented with 0.3% w/v yeast extract (THYE). Kanamycin (700 µg ml^−1^) was added to THYE to maintain selection for the *S. mutans* GMS905, GMS906 and GMS907 fusion strains. *S. mutans* GMS584 and SmuvicK, isogenic mutants of the wild-type UA159 strain, were grown in THYE supplemented with erythromycin at a final concentration of 10 µg ml^−1^, when needed.

**Table 1:** Bacterial strains and plasmids used in this study.

Strains or Plasmid	Relevant characteristics	Reference
***E. coli***		
ER2566	F- λ- *fhuA2* [lon] *ompT lacZ*::T7 gene 1 *gal sulA11* Δ(*mcrC*-*mrr*)114::IS10 R(*mcr*-73::miniTn10-TetS)2 R(zgb-210::Tn10)(TetS) *endA1* [*dcm*]	New England Biolabs
***S. mutans***		
UA159	Wild type, serotype c	ATCC 700610
GMS584	UA159-derived, *sloR*-deficient; Em^R^	[9]
SmuvicK	UA159-derived, *vicK*-deficient; Em^R^	[29]
GMS905	UA159-derived, with P*gcrR*:*cat* integrated; Kan^R^	This study
GMS906	GMS584-derived, with P*gcrR*:*cat* integrated; Em^R^, Kan^R^	This study
GMS907	SmuvicK-derived, with P*gcrR*:*cat* integrated; Em^R^, Kan^R^	This study
**Plasmids**		
pTXB1	*E. coli* expression vector with *Mxe* intein/chitin binding domain	New England Biolabs
pSG752	pTXB1 with *vicK*; Amp^R^	This study
pSG893	pTXB1 with *vicR*; Amp^R^	This study
pSG901	pTXB1 with *gcrR*; Amp^R^	This study
pSG892	pTXB1 with *comE*; Amp^R^	This study
pJL84	*S. mutans* integration vector with *mtlA-cat-phnA*; Kan^R^	[44]
pLM1	pJL84 with P*gcrR:cat* fusion; Kan^R^	This study

### Cloning and purification of VicK, GcrR & ComE

To generate a tagless version of VicK we used the Impact Kit (New England Biolabs) and generated a C-terminal VicK-Intein fusion protein by PCR amplifying the *vicK* coding sequence from *S. mutans* UA159 chromosomal DNA with oligonucleotides oSG548 and oSG550 (S1 Table). For the Intein tag to be removed with greater efficiency, the last amino acid of VicK was changed from serine to alanine using primer oSG550. The purified amplicon was digested with *Nde*I and *Sap*I and ligated into the expression plasmid pTXB1 (New England Biolabs) according to the supplier's protocol. The ligation mixture was transformed into *E. coli* ER2566 cells, selected for ampicillin resistance, and the construct was confirmed by DNA sequencing. To overexpress VicK-Intein, cells grown to mid-logarithmic phase (OD_600 nm_ 0.3–0.5) were induced with 1 mM IPTG for 3 hr at 37°C with aeration before being harvested by centrifugation (4°C, 5,000 x g, 15 min) and frozen at −20°C. To purify tagless VicK, cell pellets were thawed and resuspended in Intein column buffer (20 mM Tris-HCl, pH 8.0, 0.5 M NaCl) before lysozyme was added to 1 mg ml^−1^. After 30 min on ice, the cells were lysed by sonication and centrifuged (10,000 x g, 30 min, 4 °C). The insoluble pellet was resuspended in column buffer containing 0.5% w/v sarkosyl and incubated at 4°C for 1 h prior to centrifugation (10,000 x g, 30 min, 4°C). The resulting supernatant was diluted in column buffer to a final sarkosyl concentration of 0.05% w/v, applied to a Chitin column (New England Biolabs) and washed with Intein column buffer. To cleave VicK from the Intein tag, the column was incubated in Intein column buffer with 50 mM DTT at 4°C for 16–40 h prior to elution with additional Intein column buffer. Fractions containing tagless VicK were visualized by SDS-PAGE and the protein quantified using a Bio-Rad Protein Assay and bovine serum albumin as a standard. The purified protein was stored in 25% v/v glycerol at −80°C.

The coding sequence of *S. mutans vicR* (smu.1517) was amplified as described above for *vicK* with oligonucleotides oSG726 and oSG727 (S1 Table). To improve cleavage of the Intein tag by DTT, alanine was substituted for the *vicR* stop codon in the oSG727 primer. The *vicR* coding sequence was then cloned into pTXB1, confirmed by sequencing as described above, and transformed into ER2566 cells for protein expression. Freshly transformed cells were used to inoculate an overnight culture for VicR overexpression, which was induced with 1 mM IPTG as described above. The purification was carried out as described for tagless VicK except that the pH of the binding buffer was adjusted to pH 9.0 and the insoluble pellet was incubated with 0.65% sarkosyl for 1 hr to release the fusion protein into the soluble fraction. Pure tagless VicR was visualized by SDS-PAGE, quantified as described above and stored in 25% glycerol at −80°C.

The coding sequence of *S. mutans gcrR* (smu.1924) was amplified by PCR with primers oSG741 and oSG742 (S1 Table). To facilitate cleavage of the Intein tag by DTT, glutamine was substituted for the *gcrR* stop codon in the oSG741 primer. The *gcrR* coding sequence was digested with FauI and PvuII and then cloned into the NdeI and SapI sites on pTXB1 before being confirmed by sequencing as described above. Tagless GcrR was purified as described for tagless VicK with one exception; the pH of the column buffer was adjusted to 8.5. Pure tagless GcrR was visualized by SDS-PAGE, quantified as described above and stored in 25% glycerol at −80°C.

The coding sequence of *S. mutans comE* (smu.1917) was amplified by PCR with primers oSG691 and oSG692, cloned into pTXB1 and confirmed by sequencing as described above. Tagless ComE was purified as described for VicR except 0.7% sarkosyl was used to solubilize the fusion protein. Pure tagless ComE was visualized by SDS-PAGE, quantified as described above, and stored in 25% glycerol at −80°C.

### Phosphorylation assays

For autophosphorylation experiments, 1 µM VicK was incubated in 100 mM Tris-HCl, pH 7.5 and various metal cations were included at a final concentration of 1 mM. The phosphorylation reactions were initiated by adding 0.10 µM [γ-^32^P] ATP followed by incubation at room temperature for 15 min [40]. Reactions were stopped by adding 2X SDS sample buffer (120 mM Tris-HCl, pH 7.4, 20% v/v glycerol, 4% w/v SDS, 10% v/v ß-mercaptoethanol and 0.1% w/v bromophenol blue) and resolved on a 4–20% Tris-Glycine gel (Invitrogen) run at approximately 18.75 V cm ml^−1^ for 1.5 h. The gels were dried and scanned with a Pharos FX imaging system (Bio-Rad) and quantified using ImageQuant 5.0 (Molecular Dynamics).

For transphosphorylation reactions, 1 µM VicK was incubated for 15 minutes at room temperature with excess cold ATP (10 µM), to ensure ATP was not limiting, and 0.2 µM [γ-^32^P] ATP, 100 mM Tris-HCl pH 7.5, 50 mM NH_4_Cl, and 1 mM MgCl_2_. Then GcrR, VicR, or ComE RRs were added to 1 µM to the the VicK autophosphorylation reaction for 1 hr at room temperature. The phosphoryation reactions were diluted 1∶1 in 2x SDS loading buffer (125 mM Tris-HCl pH 7.4, 0.005% bromophenol blue, 4% SDS, 20% glycerol). A 4–20% acrylamide Tris-glycine SDS PAGE Novex gel (Invitrogen), the X-Cell SureLock Mini-Cell protein electrophoresis chamber and the Tris-glyine running buffer were pre-chilled at 4°C for ∼3 hrs. The reactions were separated by SDS-PAGE for ∼3 hrs at 150 V at 4°C. The resulting gels were dried, exposed to a phosphor screen [26] and scanned using a Typhoon phosphorimager (GE Healthcare).

For transphosphorylation reactions in the presence of MnCl_2_, 1 µM VicK was incubated in the presence of excess cold ATP (10 µM), 0.2 [γ-^32^P] ATP µM, 100 mM Tris-HCl pH 7.5, and 1 mM MnCl_2_ for 15 min at room temperature. The response regulator (1 µM) was added and the reactions were incubated for 1 hr at room temperature before being diluted 1∶1 in 2x SDS loading buffer. As described above, the buffers and gels were pre-chilled at 4°C. The transphosphorylations reactions were separated on 4–20% acrylamide Tris-glycine SDS PAGE Novex gels for 2.5 hrs at 128 V and then for 1 hr at 150 V at 4°C. The resulting gels were dried, exposed to a phosphor screen [26] and scanned using a Typhoon phosphorimager (GE Healthcare). The Phos-Tag mobility shift assay was performed as described previously [41], with the exception that a 12% gel was prepared and visualized by silver staining [42].

### DNaseI footprinting

DNaseI footprinting analysis was performed as previously described [43]. For protein concentrations used, refer to the figure legend.

### Construction of the *S. mutans* P*gcrR:cat* fusion strains GMS905, GMS906 and GMS907

The *gcrR* promoter region was amplified by PCR from the *S. mutans* UA159 chromosome using primers *gcrR*_356.FV.F and *gcrR*_356.FV.R (S1 Table). *Sac*I and *BamH*I digested amplicons were then cloned into the integration vector pJL84, which contains the *S. aureus cat* and *S. mutans phnA* and *mtlA* genes [44]. Kanamycin-resistant transformants were selected and the presence of the *gcrR* promoter region fused to *cat* was confirmed by sequencing. The resulting plasmid, pLM1, was transformed into *S. mutans* UA159, GMS584 and SmuvicK in the presence of competence stimulating peptide (CSP) according to established protocols, to generate strains GMS905, GMS906 and GMS907, respectively [9], [29], [45]. Integration of the P*gcrR:cat* fusion via allelic exchange was mediated by the *S. mutans phnA* and *mtlA* genes that are resident on the pJL84 plasmid [44]. PCR amplification and nucleotide sequencing were used to confirm the double allelic crossover event at the desired locus on the *S. mutans* chromosome.

### CAT assay

Overnight cultures of *S. mutans* GMS905, GMS906 and GMS907 were diluted 1∶10 in pre-warmed THYE and grown to an OD_600 nm_ 0.6–0.7. Cells were harvested by centrifugation, resuspended in 1 ml of 10 mM Tris-HCl at pH 7.8 and lysed by mechanical disruption in a BIO101 Savant FastPrep (Thermo Savant) for 1.5 min with intermittent cooling on ice. Unlysed cells and debris were removed by centrifugation (9,300 x g, 4 min, 4°C) and the resulting cell lysates were stored at −20°C for subsequent protein determination with a BCA protein assay kit (Pierce) and chloramphenicol acetyltransferase [35] assays. CAT assays were performed according to the method described by Shaw [46]. Briefly, a 360 µl reaction mixture consisting of 100 mM Tris-HCl pH 7.8, 0.4 mg ml^−1^ 5,5′-dithiobis(2-nitrobenzoic acid) (DTNB, (*ε*_412 nm_ = 13.6 mM^−1^ cm^−1^), and 0.1 mM acetyl-CoA was mixed with 40 µl of whole cell lysate. To monitor CAT specific activity, 0.1 mM chloramphenicol (CM) was added and absorbance readings at OD_412_ were obtained every 10 sec over a five min interval in a Synergy HT Microtiter Plate Reader (BioTek). To assess background activity, absorbance readings for wells that contained only the reaction mixture and the whole cell lysate were obtained in parallel. The rate of change due to addition of CM was determined by subtracting the background activity from the rate of change after the addition of CM. This value was divided by 0.0136 (extinction coefficient of DTNB) to yield CAT activity and then subsequently divided by the total protein concentration to express the CAT specific activity result in nM min^−1^ mg^−1^. All CAT assays were performed as three independent experiments each in triplicate.

### Quantitative real time PCR (qRT-PCR)

Overnight cultures of *S. mutans* UA159 and SmuvicK were grown to mid-exponential phase (OD_600 nm_∼0.4) in Tryptone (Bioshop) Yeast Extract Glucose medium (TYEG; 10% w/v tryptone, 5% w/v yeast extract, 17.2 mM K_2_HPO_4_, 0.5% w/v glucose, pH 7.5). Following incubation, the cells were pelleted, resuspended in TYEG at pH 7.5 or pH 5.5 and incubated for 1 h at 37°C and 5% CO_2_. The cells were harvested by centrifugation, snap frozen in liquid nitrogen and stored at −80°C. RNA was isolated from the pellets and reverse transcribed as previously described [36]. The resulting cDNAs were used as a template in qRT-PCR reactions with primers listed in S1 Table according to established protocols [36]. Gene expression was normalized to that of *S. mutans* 16SrRNA which was invariable under the experimental test conditions (data not shown). Relative expression of the target genes was calculated using results from 3 independent experiments, according to the method of Pfaffl *et al* [47].

To determine *vicR* expression in a *sloR*-deficient (GMS584) mutant vs. wild-type UA159, overnight cultures of both strains were grown in a semi-defined medium (SDM) [48] to OD_600 nm_∼0.6 before the cells were pelleted and snap frozen as described above. RNA was isolated from the pellets and reverse transcribed as previously described [4]. The expression of *vicR* was normalized to that of *gyrA*, which did not change under the experimental conditions tested (data not shown). These qRT-PCR experiments were performed in triplicate in each of three independent experiments.

## Results

### Autophosphorylation of VicK is facilitated by manganese and inhibited by iron

**Figure 1:**
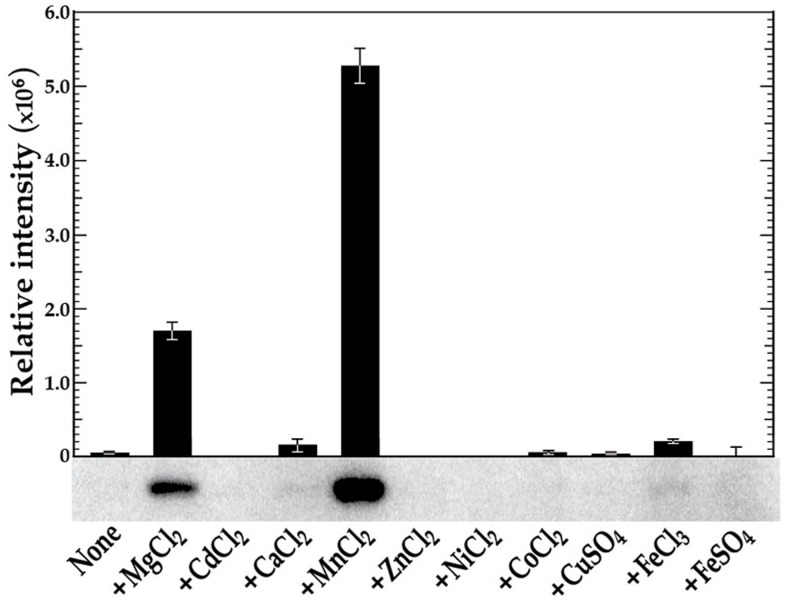
*in vitro* phosphorylation of VicK in the presence of various metal cations. VicK (1 µM) was incubated in 100 mM Tris-HCl, pH 7.5 containing 1 mM of the designated cations and 0.10 µM [γ-^32^P] ATP at room temperature for 15 minutes. The relative autophosphorylation of VicK was quantified using Image Quant 5.0 software (Molecular Dynamics) and is represented by the histogram above the scanned gel. The gels shown are representative of at least three independent experiments. Error bars represent ± std. errors of the average phosphorylation values derived from at least 3 independent experiments.

To demonstrate that VicK can undergo autophosphorylation, VicK was purified to>90% homogeneity as determined by Coomassie blue staining of SDS-PAGE gels (data not shown) and subsequently used in phosphorylation assays. Initial phosphorylation assay conditions were based on results described previously by Clausen *et al* [40]. VicK was readily phosphorylated when incubated at room temperature for 15 min in the presence of NH_4_Cl, MgCl_2_ and [γ-^32^P] ATP (data not shown). We next tested a variety of different divalent metal cations (1 mM MgCl_2_, CdCl_2_, CaCl_2_, MnCl_2_, ZnCl_2_, NiCl_2_, CoCl_2_, CuSO_4_, FeCl_3_, or FeSO_4_) in lieu of NH_4_Cl and MgCl_2_ to assess their potential impact on VicK phosphorylation. As shown in Fig. 1, VicK was able to autophosphorylate at relatively low levels in the presence of MgCl_2_, CaCl_2_, CoCl_2_, or FeCl_3_, and at significantly increased levels in the presence of MnCl_2_ (an over 3-fold increase compared to the next highest phosphorylation condition that was observed with MgCl_2_).

**Figure 2:**
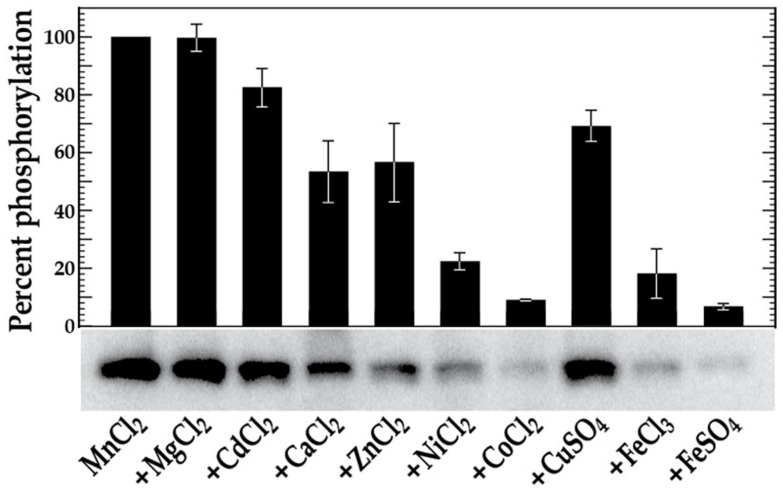
*in vitro* phosphorylation of VicK in the combined presence of Mn^2+^ and other various metal cations. VicK (1 µM) was incubated in 100 mM Tris-HCl, pH 7.5 containing 1 mM MnCl_2_ plus 1 mM of the designated cation and 0.10 µM [γ-^32^P] ATP at room temperature for 15 minutes. The relative autophosphorylation of VicK was quantified using Image Quant 5.0 software (Molecular Dynamics). The sample containing only VicK and MnCl_2_ was set at 100% for comparison and the results are shown in the histogram above the scanned gel. The gels shown are representative of at least three independent experiments. Error bars represent ± std. errors of the average phosphorylation values derived from at least 3 independent experiments.

To determine whether any of the metal cations tested above might inhibit VicK autophosphorylation in the presence of Mn^2+^, we repeated the autophosphorylation assays with reaction mixtures containing VicK, MnCl_2_ and equimolar amounts of MgCl_2_, CdCl_2_, CaCl_2_, ZnCl_2_, NiCl_2_, CoCl_2_, CuSO_4_, FeCl_3_, or FeSO_4_. CaCl_2_, NiCl_2_, and ZnCl_2_ inhibited VicK autophosphorylation by 46%, 77% and 43%, respectively, relative to the 100% phosphorylation of VicK that we observed in the presence of Mn^2+^ alone (Fig. 2). Interestingly, CoCl_2_, FeCl_3_ and FeSO_4_ had an even more pronounced effect on VicK phosphorylation in the presence of Mn^2+^, diminishing activity to 9%, 18% and 7%, respectively (Fig. 2). Importantly, the addition of either FeCl_3_ or FeSO_4_ (freshly prepared before use to limit oxidation) did not alter the overall pH of the phosphorylation reaction, consistent with a direct inhibitory effect for iron on VicK phosphorylation. Collectively, these autophosphorylation experiments demonstrate that while standard conditions (NH_4_Cl/MgCl_2_) and Mn^2+^ can readily stimulate autophosphorylation of VicK, not all divalent cations can effectively do so.

### VicK differentially transphosphorylates VicR and GcrR in the presence of manganese

**Figure 3:**
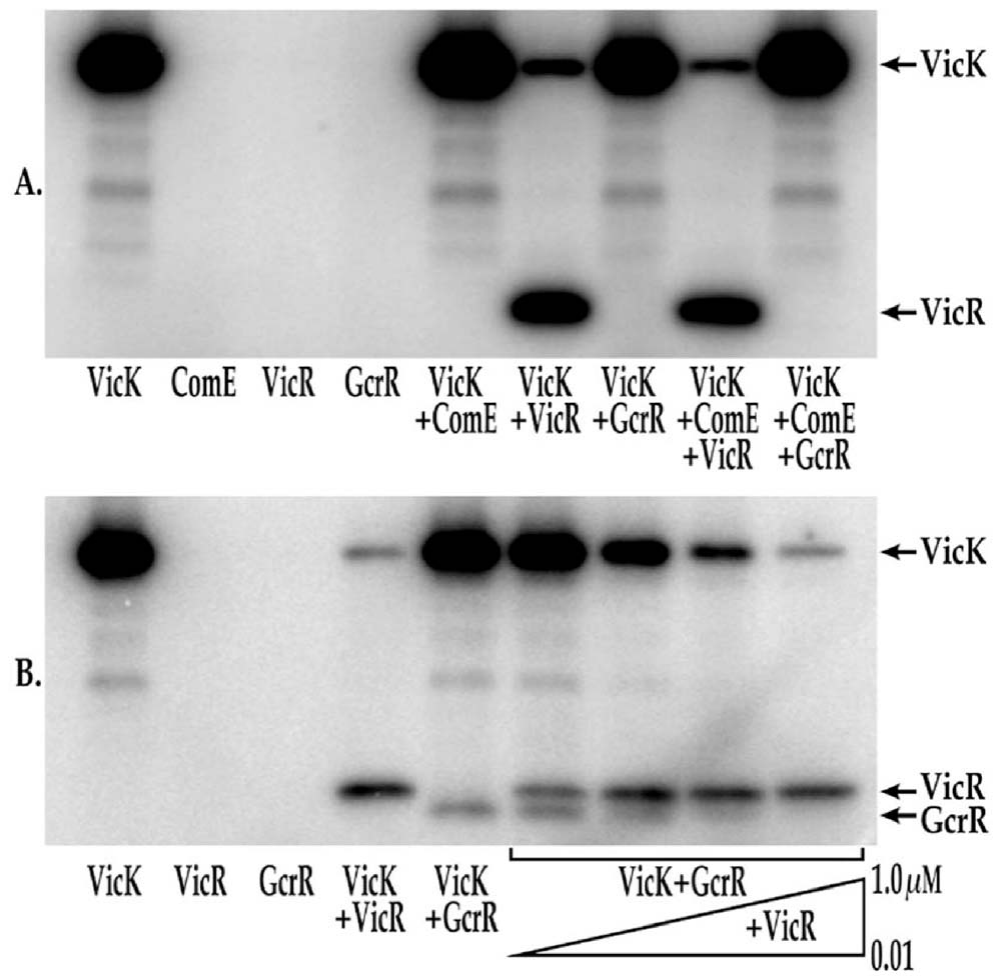
*in vitro* transphosphorylation of VicR and GcrR by VicK. A) Phosphorylation of VicR and GcrR by VicK in the presence of MgCl_2_. For each reaction 1 µM of each of the following proteins were included in the reaction: Lane 1: VicK; Lane 2: ComE; Lane 3: VicR; Lane 4: GcrR; Lane 5: VicK and ComE; Lane 6: VicK and VicR; Lane 7: VicK and GcrR; Lane 8: VicK, ComE and VicR; Lane 9: VicK, ComE and GcrR. B) Phosphorylation of VicR and GcrR by VicK in the presence of MnCl_2_. For each reaction 1 µM of each of the following proteins were included in the reaction unless otherwise indicated: Lane 1: VicK; Lane 2: VicR; Lane 3: GcrR; Lane 4: VicK and VicR; Lane 5: VicK and GcrR; Lane 6: VicK, GcrR and 0.01 µM VicR; Lane 7: VicK, GcrR and 0.02 µM VicR; Lane 8: VicK, GcrR and 0.04 µM VicR; Lane 9: VicK, GcrR and 1 µM VicR. The gels shown are a representative set of replicate gels run for each experiment.

*S. mutans* VicK's ability to transphosphorylate VicR has recently been demonstrated [41], [49], [50]. To further explore this in the presence of different divalent metal cations, we phosphorylated VicK in the presence of excess ATP, and buffer containing MgCl_2_ and NH_4_Cl and then supplemented the reaction mixture with various combinations of the VicR, ComE or GcrR responder proteins. ComE and GcrR were selected since both have overlapping pathways with the VicRK TCS based on transcriptome analysis and regulation of common phenotypes that include genetic competence, acid tolerance and biofilm formation [29], [36], [39], [51]–[53]. Despite this knowledge cross-talk with VicK has not been demonstrated. Each RR was allowed to incubate individually with VicK. As seen in Fig. 3A (lanes 5–7), VicR was the only RR that was phosphorylated under these conditions. As an additional control, each RR was added to phosphorylation reactions in the absence of VicK and no phosphorylation was observed (Fig. 3A, lanes 2–4). ComE was also added to the transphosphorylation reaction containing either VicR or GcrR. Even with the addition of ComE, only VicR was efficiently phosphorylated by VicK (Fig. 3A, lane 8). Likewise no detectable transphosphorylation was noted when GcrR and ComE were combined (Fig. 3A, lane 9).

Previous studies have shown that the GcrR regulon is manganese-responsive [4], and here we demonstrate that VicK autophosphorylation is stimulated by manganese. To date, no cross-talk has ever been observed between any of the *S. mutans* HKs tested (VicK, CiaH and LiaS) and their noncognate RRs (CiaR and LiaR) [49]; albeit these experiments were performed in the absence of divalent cations. We explored whether VicR or the GcrR orphan RR could be phosphorylated by VicK-P in the presence of manganese. The autophosphorylation and transphosphorylation reactions were allowed to proceed in the presence of excess ATP, and buffer containing MnCl_2_. As seen in Fig. 3B lane 4, VicR was phosphorylated under these conditions, although at slightly lower levels than those observed previously in transphosphorylation reactions lacking manganese. In contrast to Fig. 3A and in the presence of manganese, VicK was able to phosphorylate the non-cognate *S. mutans* response regulator, GcrR (Fig. 3B, lane 5). Importantly, neither VicR nor GcrR were capable of autophosphorylation under these conditions (Fig. 3B, lanes 2–3).

To determine whether VicK has a preference for the phosphorylation of VicR and/or GcrR in the presence of manganese, the reaction containing 1 µM GcrR was repeated in the presence of 0.01–1 µM VicR (Fig. 3B, lanes 6–9). For these experiments VicR was added to the reaction mixture after GcrR was added. The addition of even 0.02 µM VicR outcompeted GcrR as a substrate for VicK transphosphorylation (Fig. 3B, lane 7) although measurable GcrR phosphorylation persists under these conditions through VicR concentrations as high as 0.04 µM (Fig. 3B, lane 8). To demonstrate that this competition was specific for VicR and GcrR, we transphosphorylated VicR and GcrR in the presence of manganese and 1 µM ComE. As shown in S1 Fig. as much as 1 µM ComE had no effect on the phosphorylation state of either VicR or GcrR. To confirm that the bands shown in Fig. 3b are the proteins indicated, we performed transphosphorylation assays in the presence of MnCl_2_ followed by Phos-Tag mobility shift analysis [41] and silver staining with purified VicK (51.7 kDa), VicR (26.9 kDa), and GcrR (26.7 kDa) under the same conditions described above (S2 Fig.). The protein migration patterns shown in S2 Fig. correspond to those seen in Fig. 3b, demonstrating that these protein bands are the specified proteins.

### GcrR and VicR binding sites overlap at co-regulated genes

**Figure 4:**
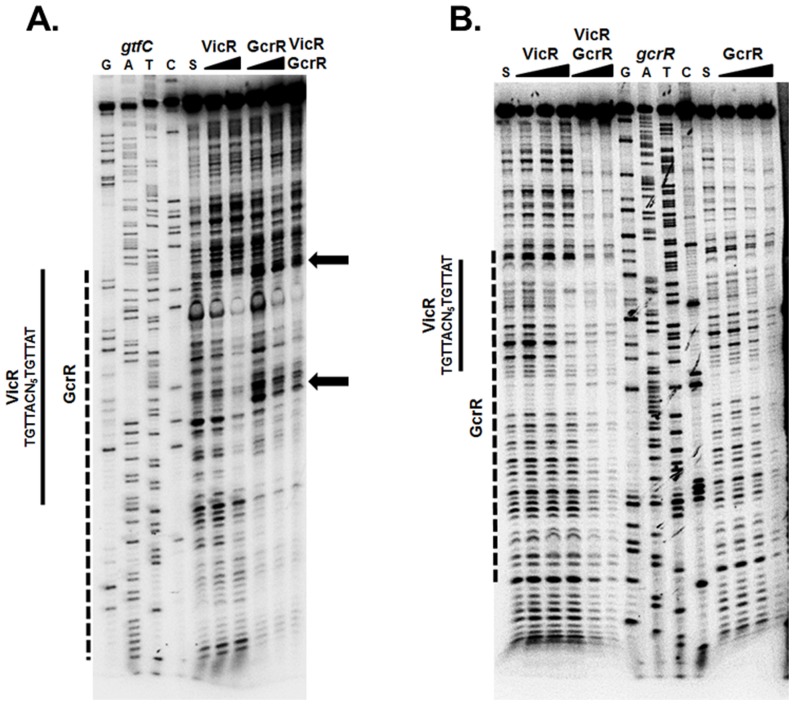
DNaseI footprinting of the *gtfC* and *gcrR* promoter regions. (A) VicR or GcrR at increasing concentrations (0.25 and 0.5 µM) or a combination of VicR and GcrR at an equimolar concentration (0.5 µM) were incubated with labeled *gtfC* DNA substrate. The S above the fifth lane indicates the DNA substrate incubated in the absence of VicR/GcrR. The arrows designate the areas of enhanced cleavage by DNaseI. (B) Labeled *gcrR* DNA substrate was incubated with increasing concentrations of VicR or GcrR (0.125, 0.25, and 0.5 µM) or a mixture of VicR and GcrR at equimolar concentrations (0.25 and 0.5 µM). The S above the first and eleventh lanes indicates the DNA substrate incubated in the absence of VicR/GcrR. The solid line represents the region of protected nucleotides by VicR and the dashed line represents the region of protection by GcrR. The VicR consensus sequence is shown to the left of the solid lines.

VicRK and GcrR regulate a number of overlapping genes including *gtfC* [2], [11], [12], [29], [52], [54]. We recently further characterized theconsensus sequence of VicR [43] and sought to gain a better understanding of the direct regulation of VicR and GcrR. Specifically, we wanted to examine the binding site boundaries of VicR and GcrR particularly since a GcrR binding consensus has proven elusive. To explore this, we used DNaseI footprinting with VicR and GcrR individually or both RRs present at an equimolar amount, with the *gtfC* promoter region as DNA substrate. As shown in Fig. 4A, VicR and GcrR independently displayed protection that overlapped the VicR consensus sequence (indicated by the solid and dashed lines respectively). Unlike VicR, the GcrR footprint exhibited enhanced cleavage (indicated by the arrows) at two specific locations. These sites correspond to the bases 5′-TGTG and 5′-GTGT that flank the VicR consensus sequence. Such “hypersensitive sites” reflect an increased accessibility of specific phosphodiester linkages to DNase I cleavage, often indicative of DNA bending. The same enhanced cleavage was also observed when VicR and GcrR were both present in the assay at an equimolar amount, whereas VicR alone displayed protection at this site (Fig. 4A, solid line). This indicates that not only do VicR and GcrR have overlapping binding sites but that GcrR under equimolar conditions exhibits greater binding affinity for *gtfC* compared to VicR.

To determine if GcrR demonstrated DNA binding dominance at another gene locus (in addition to *gtfC*), we also investigated the binding affinities of VicR and GcrR to the *gcrR* promoter region. To examine this we used DNaseI footprinting by amplifying a 160 bp fragment upstream of the *gcrR* start codon [43]. As shown previously, VicR protected a region that overlapped its consensus sequence [43] clearly seen with the highest concentration of VicR (0.5 µM) (Fig. 4B, solid line). In contrast, GcrR displayed protection that overlapped that of VicR, but the binding affinity was much stronger with protection observed at 0.25 µM (dashed line). When VicR and GcrR were present simultaneously at equimolar amounts, protected regions again overlapped but resembled that of GcrR compared to VicR, suggesting that GcrR demonstrates stronger binding affinity for the *gcrR* substrate (Fig. 4B). These results provide evidence that further integrate the functions of VicR and GcrR at select promoter regions.

### SloR positively regulates *vicRKX* expression

We previously reported the results of microarray experiments that support involvement of *S. mutans* SloR in regulating *gcrR* expression [4]. Further review of these transcriptome data revealed that expression of the *vicRKX* tricistronic operon was drastically reduced, relative to wild type, in the isogenic *sloR*-deficient GMS584 strain, consistent with a role for SloR in the transcriptional regulation of this locus. To corroborate these findings, qRT-PCR experiments were performed with cDNAs from *S. mutans* UA159 and GMS584 with *vicR*-specific primers (S1 Table). Consistent with the microarray data, *vicR* expression was over 7-fold greater in the UA159 wild-type strain as compared to *vicR* expression in the GMS584 SloR-deficient mutant (data not shown), thereby supporting a role for SloR as a positive regulator of the *vicRKX* tricistronic operon.

### Transcription of *gcrR* is subject to VicK and SloR control

**Figure 5:**
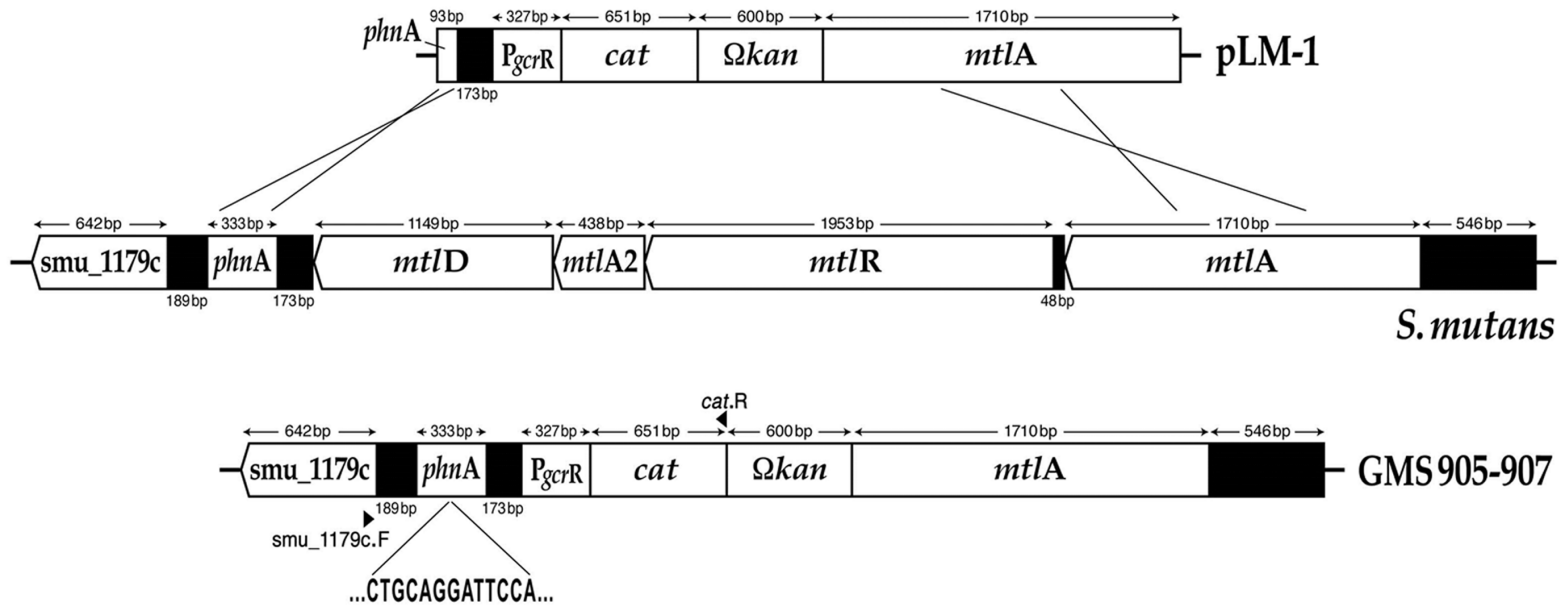
Construction of the *S. mutans* fusion strains GMS905, GMS906, and GMS907. The integration of the P*gcrR:cat* fusion that is resident on plasmid pLM1 occurred via a double cross-over event into the chromosome of *S. mutans* UA159, GMS584 and SmuvicK at the *phnA* and *mtlA* loci. Sequencing across the *S. mutans* chromosome-pLM1 junction confirmed appropriate insertion of the P*gcrR:cat* fusion.

To further elucidate the impact of VicK and SloR on *gcrR* expression, P*gcrR:cat* fusions were constructed in *S. mutans* wild-type (GMS905) and mutant (GMS906 and GMS907 which harbor *sloR* and *vicK* insertion-deletion mutations, respectively) backgrounds (Fig. 5). The resulting fusion strains were confirmed by nucleotide sequencing with Smu_1179c- and *cat*-specific primers (S1 Table). For cat assays, whole cell lysates were prepared from mid-exponential phase cultures of each strain so that CAT-specific activity could be monitored according to the spectrophotometric assay of Shaw [46]. Importantly, we observed CAT-specific activity for the wild-type GMS905 strain (5.84±1.24 nM min^−1^ mg^−1^), that was 3-fold greater than that of the GMS906 SloR-deficient derivative (1.78±0.74 nM min^−1^ mg^−1^), and 2.5-fold greater than that of the VicK-deficient strain, GMS907 (2.30±0.69 nM min^−1^ mg^−1^). These findings support a role for both SloR and VicK in modulation of the *S. mutans gcrR* gene *in vivo*.

### VicK positively regulates *gcrR* and ATR related gene transcription under low pH

VicK has previously been implicated in facilitating *S. mutans* ability to respond and adapt to low pH [39]. To further examine the role of VicK in regulating the *S. mutans* ATR, we compared the expression of known ATR genes, *atpE/A*, *ffh, radA* and *gcrR*, in the UA159 wild-type strain and a *vicK*-knockout derivative, SmuvicK [29]. Exposure of wild-type UA159 cells to a sub-lethal acid challenge (pH 5.5) resulted in over a 2-fold increase in expression at these loci compared to cells grown at pH 7.5 (data not shown). The greatest induction of gene expression in the UA159 wild-type strain was observed for *atpA* and *atpE* (>3-fold), which encode the alpha and c subunits of the F_o_F_1_ membrane-bound proton-translocating ATPases, respectively. Not surprisingly, the involvement of these ATPase subunits in the *S. mutans* ATR is well established in the literature [4], [6], [7], [55], [56].

**Figure 6:**
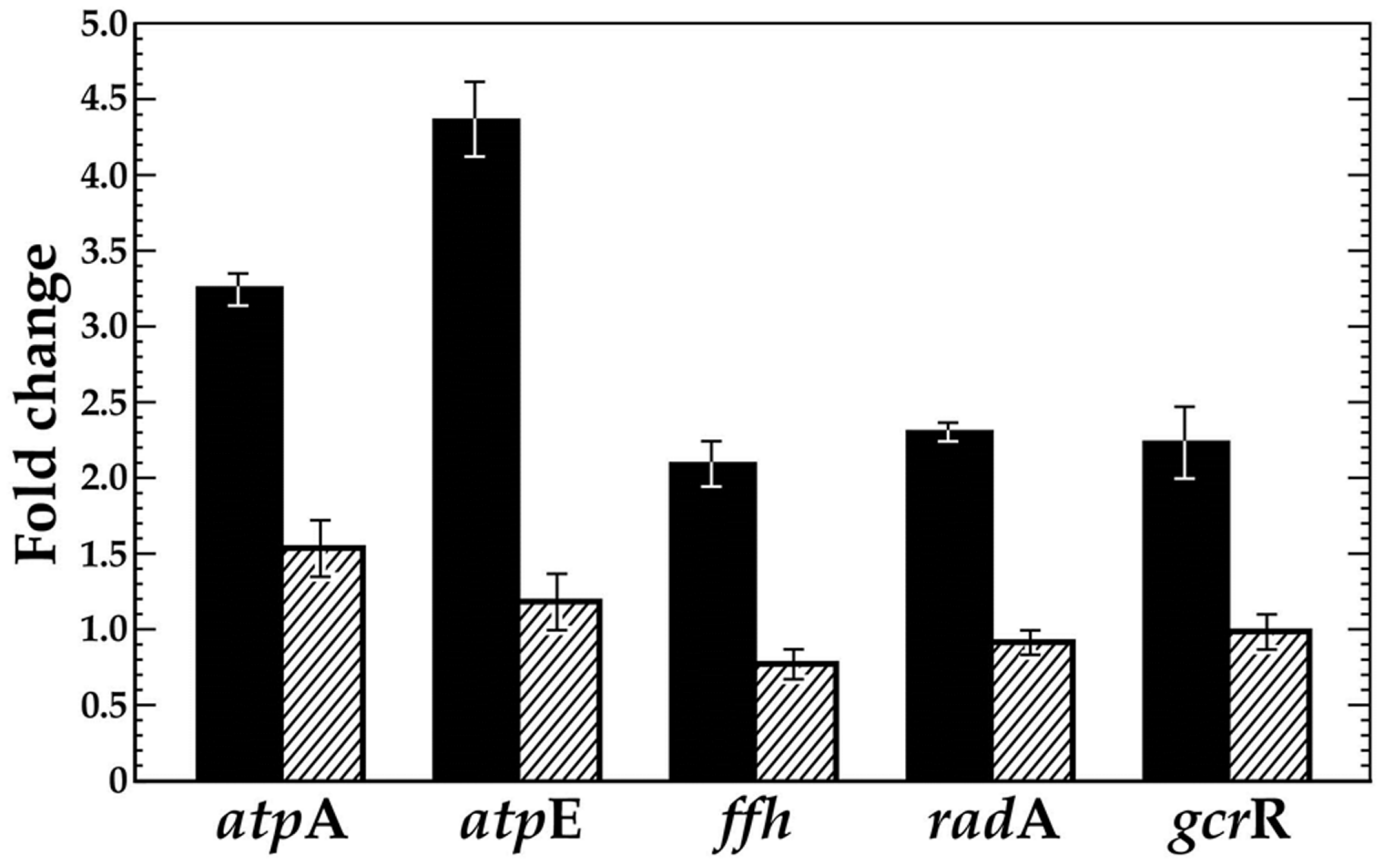
VicK has a significant impact on transcription of known ATR-related genes in *S. mutans*. qRT-PCR was performed to reveal fold-change in gene expression at pH 5.5 versus 7.5 with cDNAs derived from *S. mutans* UA159 (solid black bars) and a *vicK* insertion-deletion mutant (SmuvicK) (striped bars). Error bars represent ± std. errors of the average expression values derived from at least 3 independent experiments. Student t-tests confirm that all genes are significantly down-regulated in the VicK mutant relative to the UA159 wild-type progenitor strain (p<0.001).

In contrast, loss of VicK failed to induce any of these genes at low pH, suggesting a requirement for VicK in modulating their transcription under conditions of acid stress (Fig. 6). In addition, all of the genes were significantly down-regulated in the VicK mutant relative to that in wild type (p<0.001, with the exception of *gcrR* at pH 7.5), further supporting a positive regulatory role for VicK in their transcription (S2 Table).

## Discussion

In this report, we demonstrate that *in vitro* autophosphorylation of the *S. mutans* VicK HK is enhanced by manganese and inhibited by ferrous iron, suggesting two potential roles for VicRK in sensing manganese availability and conditions of redox. We further demonstrate that VicK, in addition to phosphorylating its cognate RR, VicR, facilitates *in vitro* cross-talk by phosphorylating the orphan RR GcrR, in the presence of manganese albeit only when VicR is present at comparatively low relative concentration. Our findings are similar to those of Guckes *et al* who report similar cross talk in the form of one HK being able to transphosphorylate specific non-cognate RRs, but only under specific conditions [57]. While VicK-facilitated phosphorylation of VicR and GcrR appears to be specific *in vitro*, it remains to be seen to what extent this phenomenon occurs *in vivo*. Interestingly, Stipp *et al* recently demonstrated that VicRK and GcrR work in concert to form structurally stable biofilms by coordinating surface biogenesis and cell division in *S. mutans* [54]. However their model did not show any direct interaction between VicK and the orphan RR GcrR. Liang *et al* identified a CovS mutant in *S. pyogenes* that retained CovR-mediated virulence gene regulation, via an unidentified alternate pathway for CovR phosphorylation [58].

Here we provide insight into the communication pathways of the *S. mutans* SloR, VicRK and GcrR regulators. Cross-regulation of GcrR by the VicK sensor kinase, may explain the overlap between the VicRK-regulon and that of GcrR, in modulating genes whose products contribute to the oxidative stress and acid tolerance responses of *S. mutans*. Work conducted previously by Dunning *et al* showed that SloR interacts directly with the *gcrR* promoter region to facilitate its expression [4]. Here we demonstrate that SloR also positively regulates expression of the *vicRKX* operon, but whether its impact on *vicRKX* transcription is direct or indirect remains to be determined. Moreover, CAT-specific activity observed in the *S. mutans cat* fusion strains support *gcrR* expression that is subject to both SloR and VicRK control.

Results of phosphorylation assays highlight a role for manganese in integrating the VicRK, GcrR and SloR regulatory pathways. Manganese is an essential micronutrient that affects *S. mutans* genes whose products are conducive to its virulence, which include those that mediate adherence and biofilm formation [59]–[62]. In fact, manganese functions as a cofactor for a *S. mutans* superoxide dismutase (SOD), which converts damaging reactive oxygen species, into less toxic substances [63], [64]. Reports in the literature describe a correlation between intracellular manganese and iron concentrations with the sensitivity of bacteria to oxidative stress [65], [66]. It has been suggested that bacteria may utilize manganese instead of ferrous iron to avoid redox conditions that are associated with Fenton chemistry [67]. This could be especially important in a biofilm environment where extracellular manganese can reach mM concentrations in the oral cavity [68].

A novel aspect of this study is our demonstration of VicK's ability to transphosphorylate GcrR under low VicR concentrations in the presence of manganese (Fig. 3B). Further, the presence of VicR causes the turnover of phosphorylated VicK, something that is not seen with other tested RRs. This effect may be important biologically as VicR-VicK interactions may activate VicK phosphatase activity reducing the net steady state levels of phosphorylated VicK; this effect seems enhanced with increasing concentrations of manganese. In contrast, we also show that iron inhibits VicK phosphorylation especially in the presence of manganese (Figs. 1 and 2). These findings are consistent with GcrR regulation by the VicRK system as well as by the metal ion-dependent SloR metalloregulator that binds manganese preferentially over iron. It is worth pointing out that we have used tagless but not necessarily native proteins for these phosphotransfer reactions. As yet to be determined, post translational modifications to each protein may be critical in each of the aforementioned reactions.

We previously described modulation of the *S. mutans* ATR by SloR with GcrR as an essential intermediary [4]. We also confirmed *sloABC* expression that is responsive to SloR and manganese concentrations that are physiological conditions likely representative of feast (>10 µM) or famine (∼0.1 µM) [4]. Our expression analysis to determine whether *sloABC* transcription was dependent on VicK revealed that loss of VicK did not significantly affect expression of *sloABC* (data not shown); suggesting that VicRK does not modulate metal ion uptake via the *sloABC* transport system. Given the *in vitro* effect of manganese on steady-state levels of GcrR and VicR phosphorylation, it is possible that VicK, like SloR, may use GcrR as an intermediary to modulate acid tolerance in *S. mutans* by responding to manganese.

GcrR exists as an orphan RR on the *S. mutans* chromosome and has 65.7% similarity to the orphan RR RitR from *Streptococcus pneumoniae*, which has a role in oxidative stress tolerance by regulating the expression of iron transport systems [69]. However, in other bacteria (such as group A streptococci where GcrR is known as CovR) GcrR is co-transcribed along with a cognate HK, CovS. Interestingly, extracellular Mg^2+^ stimulates *covRS* expression in group A streptococci (GAS) and increasing concentrations of exogenous Mg^2+^ have been shown to increase *gcrR* expression [70], [71]. Moreover, CovS can dephosphorylate (and thus inactivate) CovR in GAS under stress-inducing conditions including high temperature, low pH, high salt and iron starvation [17], [72]. It would be interesting to investigate a potential physical interaction between VicR and GcrR, and characterize the VicR- GcrR- and SloR-binding sites to validate potential cross-regulation between their respective regulons in *S. mutans*. In fact, reports in the literature supported the binding of VicR and GcrR to overlapping sequences upstream of *S. mutans gtfB/C*, encoding sucrose-dependent glucosyltransferases that are critical determinants of colonization and subsequent virulence [11], [29]. Indeed, we confirmed this hypothesis using DNaseI footprinting analysis. We demonstrated that both GcrR and VicR bind to the same regions upstream of *gtfC* and *gcrR*. When incubated together at equimolar amounts, GcrR displayed higher DNA binding affinity than VicR suggesting that *in vitro*, GcrR predominates under these conditions. Although these studies were performed using unphosphorylated forms of the RRs *in vitro*, the phosphorylation states of these RRs likely play an important role in their regulation *in vivo*. Additional studies and genomic analyses need to be performed *in vivo* to support our results.

**Figure 7:**
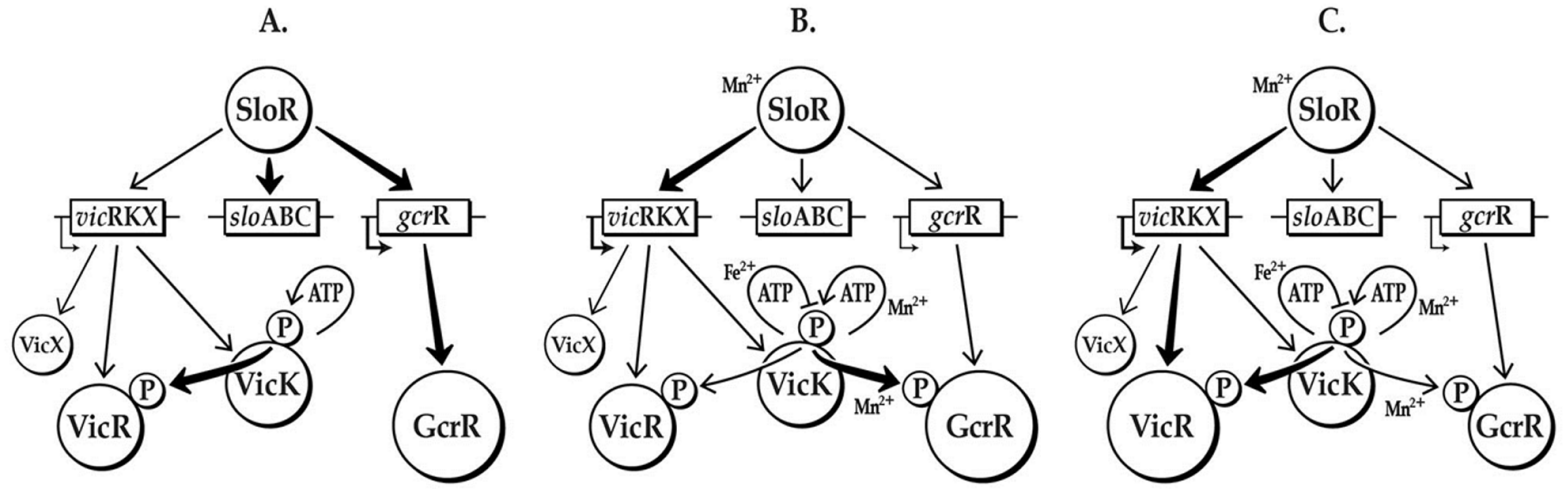
*In vitro* model of manganese-independent (A) and –dependent cross-regulation involving *S. mutans* SloR, VicRK and GcrR. A) In the absence of Mn^2+^ (approximating conditions of free-floating planktonic cells) *S. mutans gcrR* expression is de-repressed (VicR expression is not induced in the absence of Mn^2+^). Even though GcrR is the more abundant substrate, VicR is the favored species for VicK phosphorylation under these conditions. B) During this so-called “transition stage” (approximating conditions of an early biofilm) a “spike” in Mn^2+^ renders GcrR (still the predominant species) the favored substrate for phosphorylation by VicK, but only transiently. C) As the biofilm matures and Mn^2+^ concentrations increase, SloR is activated to repress *gcrR* expression, thereby reducing the availability of GcrR as a substrate. The activated SloR-Mn^2+^ complex encourages *vicR* expression, and hence VicR becomes the favored substrate for VicK phosphorylation once again.

Based on our results, we propose that it is highly likely that manganese is the common denominator for cross-communication between the VicK, GcrR, and SloR regulatory networks (Fig. 7). Both VicK and SloR activation are manganese-dependent, whereas VicK has been shown to respond to pH, oxidative and cell wall stresses [32], [33], [39], [73]. Thus, we hypothesize that VicK autophosphorylation and the subsequent transphosphorylation of VicR and GcrR (the latter in the presence of manganese) leads to modulation of numerous *S. mutans* virulence genes that facilitates bacterial survival in the presence of reactive oxygen species.

To ensure that the cross-talk between VicK and GcrR is tightly regulated, we propose that *S. mutans* regulates expression of VicR so that it may compete for phosphorylation by VicK. In the presence of manganese SloR represses *gcrR* expression and enhances VicR expression while manganese also makes GcrR a substrate for VicK transphosphorylation. In addition, when VicK, SloR or manganese are limiting there is increased expression of GcrR. It is possible that in the absence of manganese there is sufficiently more GcrR than VicR in *S. mutans* such that GcrR becomes the favored phosphoryl acceptor of VicK. Intriguingly *in vitro*, even when GcrR and VicR are present at equimolar amounts on overlapping binding sites there is evidence that GcrR binding predominates over VicR, at least in the unphosphorylated state (Fig. 4). According to our model (Fig. 7), when manganese is encountered, GcrR is phosphorylated (and thus activated) by VicK to respond to the new metal cation and possibly conditions of oxidative stress. This activation of GcrR is likely short lived due to the fact that VicR expression will increase and GcrR expression will decrease in response to manganese binding by SloR. As a result, VicR may rapidly outcompete GcrR for VicK phosphorylation, allowing for the tight regulation of crosstalk between VicK and GcrR. It is during this brief period when GcrR is phosphorylated that it alters *S. mutans* gene expression to accommodate the transition to manganese rich conditions.

Further studies are needed to determine if the phosphorylated forms of these RRs are the active or inactive forms. These additional studies are necessary to dissect the complex interactions that occur between VicK, VicR, GcrR and SloR and the larger role these regulators likely have in governing the cellular physiology of *S. mutans*.

## Supporting Information

S1 Fig **ComE has no effect on the phosphorylation state of VicR or GcrR.** Phosphorylation of VicR and GcrR by VicK in the presence of MnCl_2_ and ComE. For each reaction 1 µM of each of the following proteins were included in the reaction: Lane 1: VicK; Lane 2: ComE; Lane 3: VicR; Lane 4: GcrR; Lane 5: VicK and ComE; Lane 6: VicK, VicR; Lane 7: VicK and GcrR; Lane 8: VicK, ComE and VicR; Lane 9: VicK, ComE and GcrR. The gel shown is a representative of replicate gels run for each experiment.
(DOCX)

S2 Fig **Phos-Tag mobility shift assay of** ***in vitro*** **transphosphorylation of VicR and GcrR by VicK.** Transphosphorylation of VicR and GcrR by VicK in the presence of MnCl_2_ was performed as described in Materials and Methods followed by Phos-Tag SDS-PAGE analysis and silver staining. The protein amounts included in the reaction are indicated above each lane.
(DOCX)

S1 Table **Primers used for PCR in this study.**
(DOC)

S2 Table **Differences in ATR gene expression in Smuvick compared to wildtype.**
(DOCX)

## References

[1] Banas J (2004) Virulence properties of *Streptococcus mutans* . Front Biosci 1:1267–1277. 10.2741/1305 14977543

[2] Dmitriev A, Mohapatra SS, Chong P, Neely M, Biswas S, et al (2011) CovR-controlled global regulation of gene expression in Streptococcus mutans. PLoS One 6:e20127. 21655290 10.1371/journal.pone.0020127 PMC3105014

[3] Hanna MN, Ferguson RJ, Li YH, Cvitkovitch DG (2001) *uvrA* is an acid-inducible gene involved in the adaptive response to low pH in *Streptococcus mutans* . J Bacteriol 183:5964–5973. 11566996 10.1128/JB.183.20.5964-5973.2001 PMC99675

[4] Dunning DW, McCall LW, Powell WF Jr, Arscott WT, McConocha EM, et al (2008) SloR modulation of the Streptococcus mutans acid tolerance response involves the GcrR response regulator as an essential intermediary. Microbiology 154:1132–1143. 18375805 10.1099/mic.0.2007/012492-0

[5] Fozo EM, Quivey RG Jr (2004) The *fabM* gene product of *Streptococcus mutans* is responsible for the synthesis of monounsaturated fatty acids and is necessary for survival at low pH. J Bacteriol 186:4152–4158. 15205416 10.1128/JB.186.13.4152-4158.2004 PMC421590

[6] Hamilton IR, Svensater G (1998) Acid-regulated proteins induced by *Streptococcus mutans* and other oral bacteria during acid shock. Oral Microbiol Immunol 13:292–300. 9807121 10.1111/j.1399-302x.1998.tb00710.x

[7] Kobayashi H, Suzuki T, Unemoto T (1986) Streptococcal cytoplasmic pH is regulated by changes in amount and activity of a proton-translocating ATPase. J Biol Chem 261:627–630. 2416756

[8] Kremer BA, van der Kraan M, Crowley PJ, Hamilton IR, Brady LJ, et al (2001) Characterization of the *sat* operon in *Streptococcus mutans*: evidence for a role of Ffh in acid tolerance. J Bact 183:2543–2552. 11274114 10.1128/JB.183.8.2543-2552.2001 PMC95171

[9] Rolerson E, Swick A, Newlon L, Palmer C, Pan Y, et al (2006) The SloR/Dlg metalloregulator modulates *Streptococcus mutans* virulence gene expression. J Bacteriol 188:5033–5044. 16816176 10.1128/JB.00155-06 PMC1539950

[10] Lu L, Singh JS, Galperin MY, Drake D, Taylor KG, et al (1992) Chelating agents inhibit activity and prevent expression of streptococcal glucan-binding lectins. Infect Immun 60:3807–3813. 1500189 10.1128/iai.60.9.3807-3813.1992 PMC257393

[11] Biswas S, Biswas I (2006) Regulation of the glucosyltransferase (gtfBC) operon by CovR in Streptococcus mutans. J Bacteriol 188:988–998. 16428403 10.1128/JB.188.3.988-998.2006 PMC1347363

[12] Idone V, Brendtro S, Gillespie R, Kocaj S, Peterson E, et al (2003) Effect of an orphan response regulator on Streptococcus mutans sucrose-dependent adherence and cariogenesis. Infect Immun 71:4351–4360. 12874312 10.1128/IAI.71.8.4351-4360.2003 PMC166011

[13] Loo CY, Corliss DA, Ganeshkumar N (2000) Streptococcus gordonii biofilm formation: identification of genes that code for biofilm phenotypes. J Bacteriol 182:1374–1382. 10671461 10.1128/jb.182.5.1374-1382.2000 PMC94426

[14] Ajdic D, McShan WM, McLaughlin RE, Savic G, Chang J, et al (2002) Genome sequence of *Streptococcus mutans* UA159, a cariogenic dental pathogen. PNAS 99:14434–14439. 12397186 10.1073/pnas.172501299 PMC137901

[15] Hoch JA, Silhavy TJ (1995) Two-component signal transduction. Washington D.C.: ASM Press. 488 p.

[16] Cho KH, Caparon MG (2005) Patterns of virulence gene expression differ between biofilm and tissue communities of *Streptococcus pyogenes* . Mol Microbiol 57:1545–1556. 16135223 10.1111/j.1365-2958.2005.04786.x

[17] Dalton TL, Scott JR (2004) CovS inactivates CovR and is required for growth under conditions of general stress in Streptococcus pyogenes. J Bacteriol 186:3928–3937. 15175307 10.1128/JB.186.12.3928-3937.2004 PMC419969

[18] Graham MR, Virtaneva K, Porcella SF, Barry WT, Gowen BB, et al (2005) Group A Streptococcus transcriptome dynamics during growth in human blood reveals bacterial adaptive and survival strategies. Am J Pathol 166:455–465. 15681829 10.1016/S0002-9440(10)62268-7 PMC1602339

[19] Sumby P, Whitney AR, Graviss EA, DeLeo FR, Musser JM (2006) Genome-wide analysis of group a streptococci reveals a mutation that modulates global phenotype and disease specificity. PLoS Pathog 2:e5. 16446783 10.1371/journal.ppat.0020005 PMC1354197

[20] Virtaneva K, Porcella SF, Graham MR, Ireland RM, Johnson CA, et al (2005) Longitudinal analysis of the group A Streptococcus transcriptome in experimental pharyngitis in cynomolgus macaques. Proc Natl Acad Sci U S A 102:9014–9019. 15956184 10.1073/pnas.0503671102 PMC1150296

[21] Dalton TL, Collins JT, Barnett TC, Scott JR (2006) RscA, a member of the MDR1 family of transporters, is repressed by CovR and required for growth of *Streptococcus pyogenes* under heat stress. J Bacteriol 188:77–85. 16352823 10.1128/JB.188.1.77-85.2006 PMC1317578

[22] Graham MR, Smoot LM, Migliaccio CA, Virtaneva K, Sturdevant DE, et al (2002) Virulence control in group A Streptococcus by a two-component gene regulatory system: global expression profiling and *in vivo* infection modeling. PNAS 99:13855–13860. 12370433 10.1073/pnas.202353699 PMC129787

[23] Mattos-Graner RO, Napimoga MH, Fukushima K, Duncan MJ, Smith DJ (2004) Comparative analysis of Gtf isozyme production and diversity in isolates of *Streptococcus mutans* with different biofilm growth phenotypes. J Clin Microbiol 42:4586–4592. 15472313 10.1128/JCM.42.10.4586-4592.2004 PMC522304

[24] Goodman SD, Gao Q (2000) Characterization of the *gtfB* and *gtfC* promoters from *Streptococcus mutans* GS-5. Plasmid 43:85–98. 10610822 10.1006/plas.1999.1444

[25] Monchois V, Willemot RM, Monsan P (1999) Glucansucrases: mechanism of action and structure-function relationships. FEMS Microbiol Rev 23:131–151. 10234842 10.1111/j.1574-6976.1999.tb00394.x

[26] Fujiwara T, Hoshino T, Ooshima T, Hamada S (2002) Differential and quantitative analyses of mRNA expression of glucosyltransferases from *Streptococcus mutans* MT8148. J Dent Res 81:109–113. 11827254

[27] Li Y, Burne RA (2001) Regulation of the *gtfBC* and *ftf* genes of *Streptococcus mutans* in biofilms in response to pH and carbohydrate. Microbiology 147:2841–2848. 11577162 10.1099/00221287-147-10-2841

[28] Wexler DL, Hudson MC, Burne RA (1993) *Streptococcus mutans* fructosyltransferase (*ftf*) and glucosyltransferase (*gtfBC*) operon fusion strains in continuous culture. Infect Immun 61:1259–1267. 8454329 10.1128/iai.61.4.1259-1267.1993 PMC281356

[29] Senadheera MD, Guggenheim B, Spatafora GA, Huang Y-CC, Choi J, et al (2005) A VicR/K signal transduction system in *Streptococcus mutans* affects *gtfB/C/D, gbpB* and *ftf* expression, biofilm formation and genetic competence development. J Bacteriol 187:4064–4076. 15937169 10.1128/JB.187.12.4064-4076.2005 PMC1151735

[30] Fabret C, Hoch JA (1989) A Two-Component Signal Transduction System Essential for Growth of *Bacillus subtilis*: Implications for Anti-Infective Therapy. J Bacteriol 180:6375–6383. 10.1128/jb.180.23.6375-6383.1998 PMC107725 9829949

[31] Wagner C, de Saizieu A, Schonfeld H-J, Kamber M, Lange R, et al (2002) Genetic analysis and functional characterization of the *Streptococcus pneumoniae vic* operon. Infect Immun 70:6121–6128. 12379689 10.1128/IAI.70.11.6121-6128.2002 PMC130280

[32] Dubrac S, Bisicchia P, Devine KM, Msadek T (2008) A matter of life and death: cell wall homeostasis and the WalKR (YycGF) essential signal transduction pathway. Mol Microbiol 70:1307–1322. 19019149 10.1111/j.1365-2958.2008.06483.x

[33] Dubrac S, Boneca IG, Poupel O, Msadek T (2007) New insights into the WalK/WalR (YycG/YycF) essential signal transduction pathway reveal a major role in controlling cell wall metabolism and biofilm formation in *Staphylococcus aureus* . J Bacteriol 189:8257–8269. 17827301 10.1128/JB.00645-07 PMC2168699

[34] Ahn S-J, Burne RA (2007) Effects of oxygen on biofilm formation and the AtlA Autolysin of *Streptococcus mutans* . J Bacteriol 189:6293–6302. 17616606 10.1128/JB.00546-07 PMC1951938

[35] Deng DM, Liu MJ, ten Cate JM, Crielaard W (2007) The VicRK system of *Streptococcus mutans* responds to oxidative stress. J Dent Res 86:606–610. 17586705 10.1177/154405910708600705

[36] Senadheera MD, Lee AW, Hung DC, Spatafora GA, Goodman SD, et al (2007) The Streptococcus mutans vicX gene product modulates gtfB/C expression, biofilm formation, genetic competence, and oxidative stress tolerance. J Bacteriol 189:1451–1458. 17114248 10.1128/JB.01161-06 PMC1797355

[37] Ahn SJ, Wen ZT, Burne RA (2007) Effects of oxygen on virulence traits of *Streptococcus mutans* . J Bacteriol 189:8519–8527. 17921307 10.1128/JB.01180-07 PMC2168947

[38] Mattos-Graner RO, Porter KA, Smith DJ, Hosogi Y, Duncan MJ (2006) Functional Analysis of Glucan Binding Protein B from *Streptococcus mutans* . J Bacteriol 188:3813–3825. 16707674 10.1128/JB.01845-05 PMC1482924

[39] Senadheera D, Krastel K, Mair R, Persadmehr A, Abranches J, et al (2009) Inactivation of VicK affects acid production and acid survival of *Streptococcus mutans* . J Bacteriol 191:6415–6424. 19684142 10.1128/JB.00793-09 PMC2753040

[40] Clausen V, Bae W, Throup J, Burnham MKR, Rosenberg M, et al (2003) Biochemical characterization of the first essential two-component signal transduction system from *Staphylococcus aureus* and *Streptococcus pneumoniae* . J Mol Microb Biotech 5:252–260. 10.1159/000071077 12867749

[41] Wang C, Sang J, Wang J, Su M, Downey JS, et al (2013) Mechanistic insights revealed by the crystal structure of a histidine kinase with signal transducer and sensor domains. PLoS Biol 11:e1001493. 23468592 10.1371/journal.pbio.1001493 PMC3582566

[42] Chevallet M, Luche S, Rabilloud T (2006) Silver staining of proteins in polyacrylamide gels. Nature protocols 1:1852–1858. 17487168 10.1038/nprot.2006.288 PMC1971133

[43] Ayala E, Downey JS, Mashburn-Warren L, Senadheera DB, Cvitkovitch DG, et al (2014) A Biochemical Characterization of the DNA Binding Activity of the Response Regulator VicR from Streptococcus mutans. PLoS One 9:e108027. 25229632 10.1371/journal.pone.0108027 PMC4168254

[44] Zeng L, Burne RA (2008) Multiple sugar: phosphotransferase system permeases participate in catabolite modification of gene expression in *Streptococcus mutans* . Mol Microbiol 70:197–208. 18699864 10.1111/j.1365-2958.2008.06403.x PMC2583961

[45] Li YH, Lau PC, Lee JH, Ellen RP, Cvitkovitch DG (2001) Natural genetic transformation of *Streptococcus mutans* growing in biofilms. J Bacteriol 183:897–908. 11208787 10.1128/JB.183.3.897-908.2001 PMC94956

[46] Shaw WV (1975) Chloramphenicol acetyltransferase from chloramphenicol-resistant bacteria. Methods Enzymol 43:737–755. 1094240 10.1016/0076-6879(75)43141-x

[47] Pfaffl MW (2001) A new mathematical model for relative quantification in real-time RT-PCR. Nucleic Acids Res 29:e45. 11328886 10.1093/nar/29.9.e45 PMC55695

[48] Li YH, Lau PC, Tang N, Svensater G, Ellen RP, et al (2002) Novel two-component regulatory system involved in biofilm formation and acid resistance in Streptococcus mutans. J Bacteriol 184:6333–6342. 12399503 10.1128/JB.184.22.6333-6342.2002 PMC151940

[49] Eguchi Y, Kubo N, Matsunaga H, Igarashi M, Utsumi R (2011) Development of an antivirulence drug against *Streptococcus mutans*: repression of biofilm formation, acid tolerance, and competence by a histidine kinase inhibitor, walkmycin C. Antimicrob Agents Chemother 55:1475–1484. 21282451 10.1128/AAC.01646-10 PMC3067138

[50] Wayne KJ, Li S, Kazmierczak KM, Tsui HC, Winkler ME (2012) Involvement of WalK (VicK) phosphatase activity in setting WalR (VicR) response regulator phosphorylation level and limiting cross-talk in *Streptococcus pneumoniae* D39 cells. Mol Microbiol 86:645–660. 23013245 10.1111/mmi.12006 PMC3638944

[51] Ahn SJ, Burne RA (2007) Effects of oxygen on biofilm formation and the AtlA autolysin of Streptococcus mutans. J Bacteriol 189:6293–6302. 17616606 10.1128/JB.00546-07 PMC1951938

[52] Duque C, Stipp RN, Wang B, Smith DJ, Hofling JF, et al (2011) Downregulation of GbpB, a component of the VicRK regulon, affects biofilm formation and cell surface characteristics of Streptococcus mutans. Infect Immun 79:786–796. 21078847 10.1128/IAI.00725-10 PMC3028841

[53] Senadheera DB, Cordova M, Ayala EA, Chavez de Paz LE, Singh K, et al (2012) Regulation of bacteriocin production and cell death by the VicRK signaling system in Streptococcus mutans. J Bacteriol 194:1307–1316. 22228735 10.1128/JB.06071-11 PMC3294852

[54] Stipp RN, Boisvert H, Smith DJ, Hofling JF, Duncan MJ, et al (2013) CovR and VicRK Regulate Cell Surface Biogenesis Genes Required for Biofilm Formation in *Streptococcus mutans* . PLoS One 8:e58271. 23554881 10.1371/journal.pone.0058271 PMC3595261

[55] Belli WA, Marquis RE (1991) Adaptation of Streptococcus mutans and Enterococcus hirae to acid stress in continuous culture. Appl Environ Microbiol 57:1134–1138. 1829347 10.1128/aem.57.4.1134-1138.1991 PMC182857

[56] Quivey RG Jr, Kuhnert WL, Hahn K (2000) Adaptation of oral streptococci to low pH. Adv Microb Physiol 42:239–274. 10907552 10.1016/s0065-2911(00)42004-7

[57] Guckes KR, Kostakioti M, Breland EJ, Gu AP, Shaffer CL, et al (2013) Strong cross-system interactions drive the activation of the QseB response regulator in the absence of its cognate sensor. Proc Natl Acad Sci U S A 110:16592–16597. 24062463 10.1073/pnas.1315320110 PMC3799328

[58] Liang Z, Zhang Y, Agrahari G, Chandrahas V, Glinton K, et al (2013) A natural inactivating mutation in the CovS component of the CovRS regulatory operon in a pattern D *Streptococcal pyogenes* strain influences virulence-associated genes. J Biol Chem 288:6561–6573. 23316057 10.1074/jbc.M112.442657 PMC3585089

[59] Adkins BL, Losee FL (1970) A study of the covariation of dental caries prevalence and multiple trace element content of water supplies. N Y State Dent J 36:618–622. 5274180

[60] Kitten T, Munro CL, Michalek SM, Macrina FL (2000) Genetic characterization of a *Streptococcus mutans* LraI family operon and role in virulence. Infect Immun 68:4441–4451. 10899841 10.1128/iai.68.8.4441-4451.2000 PMC98344

[61] Paik S, Brown A, Munro CL, Cornelissen CN, Kitten T (2003) The *sloABCR* operon of *Streptococcus mutans* encodes an Mn and Fe transport system required for endocarditis virulence and its Mn-dependent repressor. J Bacteriol 185:5967–5975. 14526007 10.1128/JB.185.20.5967-5975.2003 PMC225050

[62] Spatafora G, Moore M, Landgren S, Stonehouse E, Michalek S (2001) Expression of *Streptococcus mutans fimA* is iron-responsive and regulated by a DtxR homologue. Microbiology 147:1599–1610. 11390691 10.1099/00221287-147-6-1599

[63] Jakubovics NS, Jenkinson HF (2001) Out of the iron age: new insights into the critical role of manganese homeostasis in bacteria. Microbiology 147:1709–1718. 11429449 10.1099/00221287-147-7-1709

[64] Jakubovics NS, Smith AW, Jenkinson HF (2002) Oxidative stress tolerance is manganese (Mn(2+)) regulated in *Streptococcus gordonii* . Microbiology 148:3255–3263. 12368459 10.1099/00221287-148-10-3255

[65] Archibald FS, Fridovich I (1981) Manganese, superoxide dismutase, and oxygen tolerance in some lactic acid bacteria. J Bacteriol 146:928–936. 6263860 10.1128/jb.146.3.928-936.1981 PMC216946

[66] Daly MJ (2009) A new perspective on radiation resistance based on *Deinococcus radiodurans* . Nat Rev Microbiol 7:237–245. 19172147 10.1038/nrmicro2073

[67] Anjem A, Varghese S, Imlay JA (2009) Manganese import is a key element of the OxyR response to hydrogen peroxide in *Escherichia coli* . Mol Microbiol 72:844–858. 19400769 10.1111/j.1365-2958.2009.06699.x PMC2776087

[68] Arirachakaran P, Luengpailin S, Banas JA, Mazurkiewicz JE, Benjavongkulchai E (2007) Effects of manganese on Streptococcus mutans planktonic and biofilm growth. Caries Res 41:497–502. 17992012 10.1159/000110882 PMC2820326

[69] Ong CL, Potter AJ, Trappetti C, Walker MJ, Jennings MP, et al (2013) Interplay between manganese and iron in pneumococcal pathogenesis: role of the orphan response regulator RitR. Infect Immun 81:421–429. 23184523 10.1128/IAI.00805-12 PMC3553810

[70] Chong P, Drake L, Biswas I (2008) Modulation of *covR* expression in *Streptococcus mutans* UA159. J Bacteriol 190:4478–4488. 18469111 10.1128/JB.01961-07 PMC2446802

[71] Gryllos I, Levin JC, Wessels MR (2003) The CsrR/CsrS two-component system of group A Streptococcus responds to environmental Mg^2+^ . PNAS 100:4227–4232. 12646707 10.1073/pnas.0636231100 PMC153075

[72] Froehlich BJ, Bates C, Scott JR (2009) *Streptococcus pyogenes* CovRS mediates growth in iron starvation and in the presence of the human cationic antimicrobial peptide LL-37. J Bacteriol 191:673–677. 18996992 10.1128/JB.01256-08 PMC2620807

[73] Dubrac S, Msadek T (2004) Identification of genes controlled by the essential YycG/YycF two-component system of *Staphylococcus aureus* . J Bacteriol 186:1175–1181. 14762013 10.1128/JB.186.4.1175-1181.2004 PMC344212

