# Supplementary material for: In vitro Manganese-Dependent Cross-Talk between Streptococcus mutans VicK and GcrR: Implications for Overlapping Stress Response Pathways
Source: PLoS One. 2014 Dec 23;9(12):e115975. doi: 10.1371/journal.pone.0115975 (PMC4275253; doi:10.1371/journal.pone.0115975)
Supplement: S1 Fig — ComE has no effect on the phosphorylation state of VicR or GcrR. Phosphorylation of VicR and GcrR by VicK in the presence of MnCl2 and ComE. For each reaction 1 µM of each of the following proteins were included in the reaction: Lane 1: VicK; Lane 2: ComE; Lane 3: VicR; Lane 4: GcrR; Lane 5: VicK and ComE; Lane 6: VicK, VicR; Lane 7: VicK and GcrR; Lane 8: VicK, ComE and VicR; Lane 9: VicK, ComE and GcrR. The gel shown is a representative of replicate gels run for each experiment. (DOCX) [file pone.0115975.s001.docx]

**Figure S1**


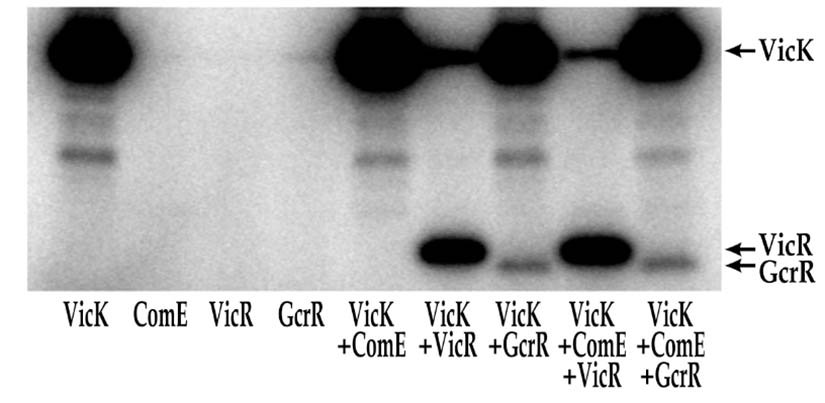


**Fig. S1.** **ComE has no effect on the phosphorylation state of VicR or GcrR.**  Phosphorylation of VicR and GcrR by VicK in the presence of MnCl_2_ and ComE. For each reaction 1 µM of each of the following proteins were included in the reaction: Lane 1: VicK; Lane 2: ComE; Lane 3: VicR; Lane 4: GcrR; Lane 5: VicK and ComE; Lane 6: VicK, VicR; Lane 7: VicK and GcrR; Lane 8: VicK, ComE and VicR; Lane 9: VicK, ComE and GcrR. The gel shown is a representative of replicate gels run for each experiment.
