# Supplementary material for: In vitro Manganese-Dependent Cross-Talk between Streptococcus mutans VicK and GcrR: Implications for Overlapping Stress Response Pathways
Source: PLoS One. 2014 Dec 23;9(12):e115975. doi: 10.1371/journal.pone.0115975 (PMC4275253; doi:10.1371/journal.pone.0115975)
Supplement: S2 Fig — Phos-Tag mobility shift assay of in vitro transphosphorylation of VicR and GcrR by VicK. Transphosphorylation of VicR and GcrR by VicK in the presence of MnCl2 was performed as described in Materials and Methods followed by Phos-Tag SDS-PAGE analysis and silver staining. The protein amounts included in the reaction are indicated above each lane. (DOCX) [file pone.0115975.s002.docx]

**Figure S2**


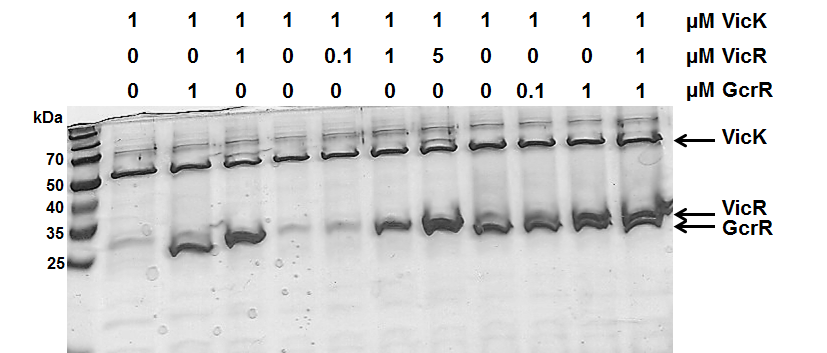


**Fig. S2. Phos-Tag mobility shift assay of *in vitro* transphosphorylation of VicR and GcrR by VicK.** Transphosphorylation of VicR and GcrR by VicK in the presence of MnCl_2_ was performed as described in Materials and Methods followed by Phos-Tag SDS-PAGE analysis and silver staining. The protein amounts included in the reaction are indicated above each lane.
