# Supplementary material for: In vitro Manganese-Dependent Cross-Talk between Streptococcus mutans VicK and GcrR: Implications for Overlapping Stress Response Pathways
Source: PLoS One. 2014 Dec 23;9(12):e115975. doi: 10.1371/journal.pone.0115975 (PMC4275253; doi:10.1371/journal.pone.0115975)
Supplement: S1 Table — Primers used for PCR in this study. (DOC) [file pone.0115975.s003.doc]

Table S1. Primers used for PCR in this study

| **Primer** | **Nucleotide sequence^*^** |
| --- | --- |
| **Cloning**  oSG548  oSG550  oSG691  pSG692  oSG726  oSG727  oSG741  oSG742  *gcrR*_356.FV.F *gcrR*_356.FV.R  **Sequencing**  Smu_1179c.F  *cat*.R  **qRT-PCR**  16SrRNA-FOR  16SrRNA-REV  ffh-FOR  ffh-REV  radA-FOR  radA-REV  atpA-FOR  atpA-REV  atpE-FOR  atpE-REV  gcrR-FOR  gcrR-REV  gyrA-RT-F  gyrA-RT-R  vicR forward  vicR reverse | 5’ CGC**CATATG**ACTAATGTGTTTGAATC 3’  5’ GGTGGTT**GCTCTTC**CGCATGCTTCGTCTTCATC 3’  5’ggtggt**catatg**atttctatttttgta 3’  5’-ggtggtt**gctcttc**cgcattttgctctcctttg-3’  5’ ggtggt**catatg**aagaaaattctaatc 3’  5’ggtggtt**gctcttc**cgcacgcatcgtagcttttcatgtaataaccaac3’  5’ GCG**CCCGCGACA**TATGGCTAAGGACA 3’  5’ CGC**CAGCTG**ATTATTTTCGCGAATG 3’  5' C**GAGCTC**GCTTCAAAGCTTCAAATTGTTC 3’  5' CG**GGATCC**ATACTCCTCAACAAAACTCTAAC 3’  5' GACAGTTCAACTTGCTCTACTC 3'  5' TTATAAAAGCCAGTCATTAGG 3'  5′ CTTACCAGGTCTTGACATCCCG 3′  5′ ACCCAACATCTCACGACACGAG 3′  5' AAGGTAAGCAAGTCTCCCATTC 3'  5' TCCGTCAAATCACTGGAAAAC 3'  5' TGGTGAAGAATCAGCGGAGC 3'  5' TCAATCTCAGCACGAATACTTTGC 3'  5' CGCAACAATGGTCACAAG 3'  5' GGAGTTTTGGCTAATCAC 3'  5' CTTACAACTTCAGATTTAGCAG 3'  5' AGAGATTCAGTCCCTATTATC 3'  5' ACCAGAGATGGACGGGTATG 3  5' CACGATAGGTAGTGTCATTTTTAGAAG 3'  5’ ATTGTTGCTCGGGCTCTTCCAG 3’  5’ ATGCGGCTTCTCAGGAGTAACC 3’  5' TTGAATCCGCAGTGGCTGAGG 3'  5' CCTGACCTGTGTGTGTCGCTAAGTG 3' |

^*^Bold indicates restrictions sites used for cloning.
