# Supplementary material for: In vitro Manganese-Dependent Cross-Talk between Streptococcus mutans VicK and GcrR: Implications for Overlapping Stress Response Pathways
Source: PLoS One. 2014 Dec 23;9(12):e115975. doi: 10.1371/journal.pone.0115975 (PMC4275253; doi:10.1371/journal.pone.0115975)
Supplement: S2 Table — Differences in ATR gene expression in Smuvick compared to wildtype. (DOCX) [file pone.0115975.s004.docx]

**Table S2: Differences in ATR gene expression in Smuvick**

**compared to wildtype.**

| **Gene** | **Fold change** | **SD** | **P value** |
| --- | --- | --- | --- |
| **pH 7.5** |  |  |  |
| *atpA* | -2.4 | ± 1.0 | p<0.001 |
| *atpE* | -2.6 | ± 2.0 | p<0.001 |
| *ffh* | -2.9 | ± 0.8 | p<0.001 |
| *radA* | -1.9 | ± 0.4 | p<0.001 |
| *gcrR* | -1.3 | ± 1.0 | NS |
| **pH 5.5** |  |  |  |
| *atpA* | -4.2 | ± 1.1 | p<0.001 |
| *atpE* | -5.5 | ± 1.8 | p<0.001 |
| *ffh* | -5.6 | ± 5.9 | p<0.001 |
| *radA* | -4.1 | ± 1.1 | p<0.001 |
| *gcrR* | -3.4 | ± 1.1 | p<0.001 |

ATR: Acid tolerance response, SD: standard deviation,

NS: not significant
